# Comparison of Efficacy of Self-Expandable Metallic Stent Placement in the Unresectable Esophageal Cancer Patients

**DOI:** 10.1155/2017/2560510

**Published:** 2017-07-27

**Authors:** Masaya Uesato, Yasunori Akutsu, Kentarou Murakami, Yorihiko Muto, Akiko Kagaya, Akira Nakano, Mizuho Aikawa, Tomohide Tamachi, Takahiro Arasawa, Hiroyuki Amagai, Yasuhide Muto, Hisahiro Matsubara

**Affiliations:** Department of Frontier Surgery, Chiba University Graduate School of Medicine, Chiba-shi, Chiba 260-8670, Japan

## Abstract

This is a retrospective study to evaluate the prevention of complications of metallic stent placement in patients with unresectable advanced esophageal cancer. A total of 87 patients were treated with 4 types of metal stents in the esophagus over a period of 18 years. Stent placement was technically successful. The most common prior treatment was chemoradiotherapy. There were no significant differences in the rate of patients with no complications among the prior treatments. Approximately, 30% of patients had the most common chest pain in complications. Stent placement within one month after the completion of chemoradiotherapy should be avoided for the prevention of the chest pain. There was no significant difference in the rate of patients with no complications by lesion location. The rate of no complications was higher for the Niti-S stent than the Gianturco Z-stent or Ultraflex stent. Of note, no complications were noted for the Niti-S ultrathin stent at all. Among cases of stent-related death, the most common type of complication was respiratory disorder caused by the stent that seems to be thick and hard. Therefore, the stent with thin and flexible characteristics like the Niti-S ultrathin stent will solve the various problems of esophageal stent placement.

## 1. Introduction

Self-expandable metallic stent (SEMS) placement is used widely for the palliative treatment of unresectable malignant esophageal stricture [1–3]. Complications were reported at rates ranging from 36%–40% [4]. In particular, major complications such as hemorrhaging, perforation, fistula, a fever, and aspiration pneumonia have been reported to occur in 22% of cases [4]. Although several researchers [5–9] have reported that previous radiotherapy (RT) and chemotherapy are associated with an increased risk of life-threatening complications, other researchers [10, 11] have reported no relationship of life-threatening complications and these therapies.

We have placed SEMSs in patients with esophageal cancer over a period of approximately 20 years. Therefore, we examined their data, retrospectively, and would like to recommend the prevention of complications based on many experiences of metallic stent placement in patients with unresectable esophageal cancer.

## 2. Materials and Methods

### 2.1. Patients

A total of 87 patients were treated with a metal stent in the esophagus from December 1997 to April 2015 at the Chiba University Graduate School of Medicine. Data from these patients were collected retrospectively. The poststented follow-up period was ranged 2–153 days, mean 59.3 days, and median 52 days. A tumor was considered inoperable if the patient had distant metastasis, local tumor infiltration in neighboring organs, or a poor health condition. The expected average prognosis was around three months. We excluded patients who were scheduled to undergo future treatment, were not expected to be able to eat again, had recurrent nerve paralysis, or had a tumor growth within 2 cm of the upper esophageal sphincter. Furthermore, because the esophagus after stent insertion does not move peristaltically, we excluded any patients who were unable to sit up.

All patients were evaluated before stent placement and at discharge. Furthermore, we performed evaluations by interviewing the patients' family about the following items: (1) ability to eat and/or swallow (graded as 0, normal swallowing; 1, able to swallow some but not all solids; 2, able to swallow semisolids; 3, able to swallow fluids only; 4, unable to swallow fluids [12]); (2) specific symptoms, such as chest pain, nausea, hiccup, and reflux; and (3) complications, such as recurrent dysphagia, stent migration, hemorrhaging, perforation, airway narrowing, aspiration pneumonia, and respiratory failure. Chest pain was defined as patients begin to use analgesics within a few days after stent placement or increase their use. The relationship of the specific symptoms, complications and prior treatment, location of lesion, and the types of SEMS was examined. We also evaluated the number of stent-related deaths within one month after placement.

### 2.2. Stent Placement and Choice

During stent insertion, all patients were consciously sedated with midazolam and pentazocine. The upper tumor margin was marked with an endoscopic hemoclip confirmed by radioscopy. A guidewire was inserted into the esophagus. A catheter for endoscopic retrograde cholangiopancreatography was inserted over the guidewire. The length of stricture was measured under radioscopy. The stents were advanced over the guidewire into the esophagus. The stricture was never dilated with a dilator such as a balloon. If the delivery system of the stent was unable to pass the tumor, the stent was covered with a polyvinyl chloride tube to make the axis stronger.

**Figure 1:**
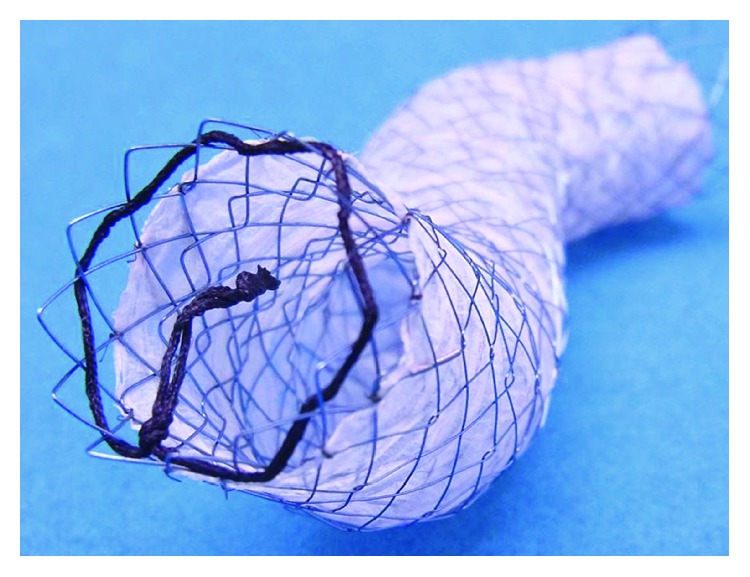
Partially covered Niti-S stent (Taewoong Medical) 10 cm long with a diameter of 16 mm. The partially covered stent is uncovered at both ends over a distance of 0.5 cm. This stent is able to maintain a three-dimensional form.

**Figure 2:**
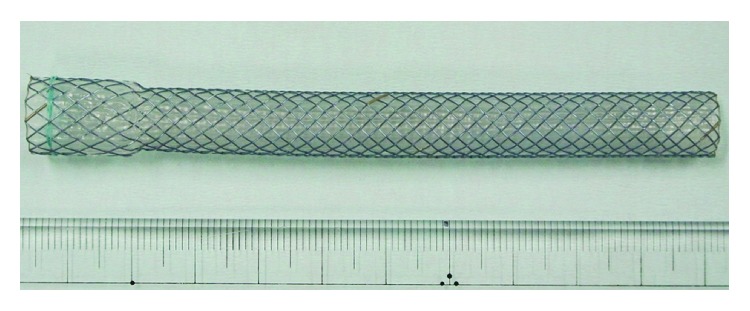
Fully covered Niti-S ultrathin stent (Taewoong Medical) 10 cm long with diameters of 12 mm (proximal) and 10 mm (middle-distal).

Four types of stent were used. We actually confirmed the feel of many kinds of stents released, and the tenderest stent was chosen in the time. The characteristics of the Gianturco Z-stent (Z stent), Ultraflex stent (UF stent), Niti-S stent (NS stent) (Figure 1), and Niti-S ultrathin stent (NSu stent) (Figure 2) are shown in Table 1. The NSu stent was inserted into the cervical or upper thoracic esophagus, which overlapped with trachea.

### 2.3. Statistical Analyses

Fisher's exact test was used to evaluate the differences in the proportions between the groups after chemoradiotherapy (CRT). The statistical analyses were conducted using the SPSS 15.0 software package (SPSS Inc., Chicago, IL, USA). *P* values of less than 0.05 were considered to be statistically significant.

## 3. Results

The patient characteristics are outlined in Table 2. Of the 87 analyzed patients, 79 (90.8%) were male, and the median age was 66.9 years (range 39–87 years). The reasons for stent placement were stenosis/fistula: 63 (72.4%)/24 (27.6%) patients. The most common prior treatment was CRT, in 51 patients (58.6%). Stent placement was technically successful in 91/93 times (97.8%) among 87 patients. The stent-in-stent after regrowth was performed in four patients. Twice stent-in-stent was done in one patient.

The changes in the oral alimentation status of the patients are shown by dysphagia score in Table 3. The mean improvement score (preplacement dysphagia score–postplacement dysphagia score) was 1.64. The best improvement score was 2, for the NS stent. The relationship between complications and prior treatment is shown in Table 4. There were no significant differences in patients with no complications by prior treatment except for RT. Approximately, 30% of patients had the most common chest pain in complications. Only one case of a serious complication after the CRT was noted (perforation). The relationships of chest pain and the period from CRT (60 Gy) completion to stent insertion are shown in Table 5. Chest pain occurred in more patients within 30 days of stent placement than above 61 days after placement (*P* = 0.063). The relationships between complications and the lesion location are shown in Table 6. There were no significant differences in patients with no complications by the lesion location. Gastroesophageal reflux was confirmed in 5 cases (17.2%) in the lower thoracic and abdominal esophagus. The relationships of the complications and the type of SEMS are shown in Table 7. The rate of no complication with NS stents (77.8%) was higher than that with Z (41.2%) or UF stents (43.4%). Of note, there were no complications with NSu stents. The stent-related mortalities within one month are shown in Table 8. The most frequent type of complication was respiratory disorder.

## 4. Discussion

Although this study is retrospective, a lot of countermeasures have been revealed for safe and effective esophageal stent placement. Stent placement within one month after the completion of CRT should be avoided for the prevention of the chest pain. Death soon after stent placement had a strong relationship with respiratory complications. To prevent such complications, an ultrathin stent might be best.

SEMS placement is used widely for the palliative treatment of unresectable malignant esophageal stricture [1–3]. Major complications such as hemorrhaging, perforation, fistula, a fever, and aspiration pneumonia occurred in 22% of subjects [4]. Therefore, the implementation of esophageal SEMSs has been sluggish. However, we have achieved good results without many serious complications at our institution. Chest pain is the most frequent complication, accounting for about 30% of patients. There is no predictive method of chest pain, but the preventive prescription of analgesia is performed now as in other reports [13].

**Figure 3:**
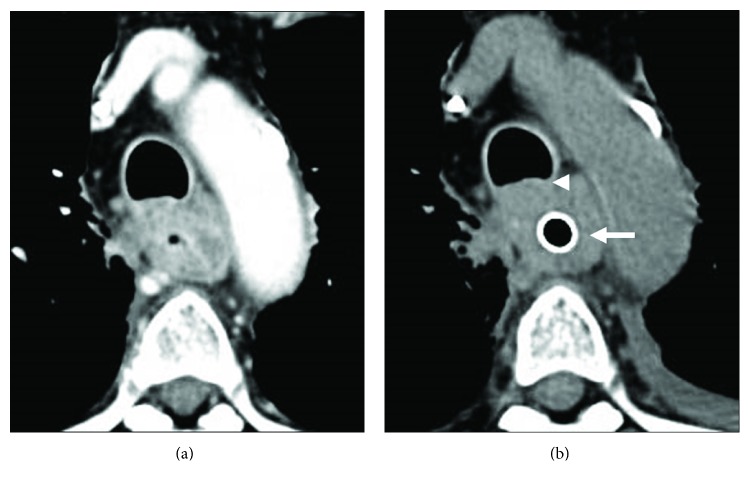
(a) CT findings before stent placement. Two months after chemoradiotherapy, the tumor located in the middle thoracic esophagus had invaded the trachea. (b) CT findings after Niti-S ultrathin stent placement (arrow). The membranous portion of the trachea did not transform (arrowhead).

The stent-related deaths occurred in patients who died within one month after insertion. All cases including a patient that occurred pneumothorax after esophageal perforation had respiratory disorders. Two patients who had complications just after stent insertion used the Z stent with high radial and axial forces. Moreover, two cases using Z stent suffered from respiratory failure. The rapid increase of the right pleural effusion was pointed out. Therefore, there may have been damage to the thoracic duct. Generally, stents with strong expansion and a wide lumen are deemed suitable. However, such stents can cause many severe complications [4]. In our study, the ratio of stent-related complications was low, due in part to our use of NS stents. NS stents have several characteristics that make them promising, such as their ultrathin size, low radial force [14], moderate axial force [14], and thin delivery system. Of note, no other stent had a low axial force in our study. If the tumor has infiltrated neighboring organs, the esophageal axis often cannot maintain a straight line. Figure 1 shows that NS stents can maintain axial flexure. In addition, NS stents do not apply high power to such flexure. Therefore, we generally opt for the NS stent. Two cases had tumors located in the cervical or upper-middle thoracic portion and died of airway narrowing. The outside diameter of the general esophageal SEMSs is known to be 18 mm. Large-diameter stents reduce the risk of recurrence dysphagia due to stent migration, tissue overgrowth, or food obstruction. Increasing the diameter, however, increases the risk of stent-related complications [4]. Questions remain as to whether or not this size is appropriate in Japanese patients. If a stent is inserted in the cervical or upper thoracic, or middle thoracic portion, we must consider the risk of the trachea being pressed. The distance from the trachea to the vertebra should be confirmed by computed tomography beforehand. We further suggest that NSu stents be used (Figure 2). We imported NSu stents into Japan for the first time and began using them in our patients.  Figure 3(a) shows a case of a tumor in the upper thoracic portion. After the placement of a 10 mm NSu stent, the membranous portion of the trachea did not transform (Figure 3(b)). A thin and soft stent like NSu stent will reduce the risk of compressing the tracheal membranous portion and might be best to prevent respiratory complications. Patients with esophageal cancer stenosis or fistel retain the hope of continuing to eat normally. However, the dysphagia improvement score with NSu stents was 1.33. Therefore, such patients will be not able to eat all meals in the usual way. However, the salivary deglutition is improved following the procedure, and the patients' quality of life is markedly improved as well. Gastroesophageal reflux was observed in 5 cases (5/29; 17.2%) of lower thoracic and abdominal portion. One of these patients died of aspiration pneumonia. Unfortunately, no stent with an antireflux valve has been marketed. This complication can now be avoided [15].

It might be very important not to dilate the stenosis using a balloon before stent insertion. When an esophageal stent is inserted, such as a UF stent, prior dilation is generally performed with a 10 to 18 mm balloon [16, 17]. However, several researchers [4, 18] recommend that prior dilation to more than 12 mm or 7-8 mm in a single session and too-quick dilation be avoided. We experienced one fatal case due to esophageal perforation caused by 8 mm balloon dilation [19, 20]. Therefore, we deemed expansion by a balloon unfavorable and did not always perform it with UF and NS stenting. If the delivery system could not pass through a severe stenosis, an overtube was used to cover the system to prevent axial arcuation and enable us to pass the system. Its flexibility makes the system difficult to pass through the stenosis. There is a report [21] that revealed the usefulness of stent placement using an overtube for malignant gastroduodenal obstructions. Our idea involves the same method of preventing flexure in the greater curvature as in that study. On the placement, however, we had two unsuccessful patients. The first patient was inserted the next stent into the mesh portion of the prior stent during the stent-in-stent. The second patient was a failure because we could not recognize that the guidewire was inserted outside the wall.

Regardless of previous treatment, about 50% of patients who underwent esophageal stent placement suffered complications in our study. The use of SEMSs in patients with prior RT is strictly limited [5–9]. Although RT to the esophagus has acute and late toxicity, the late effects of radiation are believed to be due to inflammation and scar formation within the esophageal musculature [22]. The late effects of RT are seen three or more months after completion of RT, with a median time to onset of six months [23, 24]. Iwasaki et al. [25] reported that the late effects of esophageal RT have an important role in the occurrence of severe stent-related complications. In our study, the mean observation period was 59.3 days. Furthermore, our criterion regarding stent placement is a prognosis of around three months, which may have prevented our experience of a high rate of late effects. In the acute phase, chest pain occurred in more patients with less than 30 days between CRT completion and stent insertion than in those with more than 61 days' interval. The acute effects of radiation are thought to be due to temporary inflammation such as ulcer and erosion. Moreover, the interaction between radiation and chemotherapy affects the severity of esophagitis [22]. Therefore, stent placement within one month after the completion of CRT should be avoided.

This study has several limitations. It was a retrospective study performed in a single institution. The stent placement was done for only patients with a prognosis of three months or less. In an additional investigation, 63.2% (55/87) of patients could be followed and the 3-month survival rate was 34.5% (19/55). We believe that the expected prognosis was not significantly different from the reality. However, the applications of esophageal SEMS placement should be further clarified in large, prospective studies.

## 5. Conclusions

In this study, a lot of problems associated with metallic stents in patients with unresectable advanced esophageal cancer were revealed. However, we suggest that the stent with thin and flexible characteristics like the Niti-S ultrathin stent will solve the various problems of esophageal stent placement. If the stent placement in patients with prior CRT is performed, the placement within one month after the completion of CRT should be avoided for the prevention of the chest pain.

## Figures and Tables

**Table 1:** Self-expandable metallic stent characteristics.

Characteristics	Gianturco Z-stent	Ultraflex stent	Niti-S stent
Products	Cook Medical Co., Denmark	Boston Scientific Co., Ireland	Taewoong Medical Co., Korea
Material	Stainless	Nitinol	Nitinol
Membrane material	Polyethylene	Polyethylene	Polytetrafluoroethylene
Outside diameter (mm)	18	17	18, 16, 10 (NSu stent)
Major axis (mm)	100, 120, 140	100, 150	80, 100, 120, 150
Delivery system diameter (Fr)	24	24	16.5
Shortening (%)	None	70	70
Softness	−	++	+++
Extended force	+++	++	+
Visibility	Good	Good	Good
Justification	Impossible	Possible	Possible
Removed	Impossible	Possible within 2 weeks	Possible within 2 weeks

+: mild; ++: moderate; +++: severe.

**Table 2:** Patient and tumor characteristics.

Characteristics	
Gender (*n*)	
Male	79
Female	8
Age (*n*)	
Range	39–87
Median	66.9
Tumor site (*n*)	
Ce, Ut	12
Mt	47
Lt, Ae	28
Prior treatment^※^	
None	14
CRT	51
RT	1
CT	23
Others	16
Reason for unresection^※^	
T4^※※^	45
N3, N4^※※^	24
M1^※※^	18
Poor condition	23
Rejection	1
Reason for insertion	
Stenosis	63
Fistula	24

Ce: cervical esophagus; Ut: upper thoracic esophagus; Mt: middle thoracic esophagus; Lt: lower thoracic esophagus; Ae: abdominal esophagus; CRT: chemoradiotherapy; RT: radiotherapy; CT: chemotherapy. ^※^There is some overlap; ^※※^Japanese Classification of Esophageal Cancer [26].

**Table 3:** Improvement in the dysphagia score (mean ± SD).

Stent	Number of times	Prescore	Postscore	Improvement score
Z	17	3.65 ± 0.49	2.35 ± 1.22	1.3
UF	53	3.21 ± 0.95	1.45 ± 1.2	1.76
NS	18	4 ± 0.57	2 ± 1.11	2
NSu	3	4	2.67 ± 0.58	1.33
Total	91	3.43 ± 0.82	1.79 ± 1.25	1.64

Z: Cook-Z stent; UF: Ultraflex stent; NS: Niti-S stent; NSu: Niti-S ultrathin stent; Improvement score = prescore minus postscore.

**Table 4:** Relationships between complications and prior treatment (*n* = 91 times, number (%)).

	None *n* = 13	CRT^※^ *n* = 51	RT *n* = 1	CT^※^ *n* = 23	Others^※^ *n* = 16
Chest pain^※^	2 (15.4)	15 (29.4)	1 (100)	8 (34.8)	4 (25.0)
Nausea^※^	0	1 (2.0)	0	1 (4.3)	0
Hiccup^※^	0	1 (2.0)	0	1 (4.3)	0
Gastroesophageal reflux^※^	2 (15.4)	0	0	2 (8.7)	0
Recurrent dysphagia^※^	2 (15.4)	1 (2.0)	0	1 (4.3)	1 (6.3)
Stent migration^※^	0	1 (2.0)	0	0	0
Hemorrhaging^※^	0	0	0	1 (4.3)	0
Perforation^※^	1 (7.7)	1 (2.0)	0	0	0
Airway narrowing^※^	1 (7.7)	0	0	0	1 (6.3)
Aspiration pneumonia^※^	0	0	0	1 (4.3)	0
Respiratory failure^※^	1 (7.7)	1 (2.0)	0	0	0
None	6 (46.2)	27 (52.9)	0	13 (56.5)	9 (56.3)

CRT: chemoradiotherapy; RT: radiotherapy; CT: chemotherapy. ^※^There is some overlap.

**Table 5:** Relationships of the chest pain and the period from chemoradiotherapy (60 Gy) completion to stent insertion (*n* = 20 patients).

Period from CRT to insertion (days)	1–30	31–60	61 and above
Gender			
Male	6	4	10
Female	0	0	0
Age			
Median	64.8	64.6	65.9
Tumor location			
Ce, Ut	2	0	2
Mt	3	3	5
Lt, Ae	1	1	3
Reason for intubation			
Stenosis	5	2	7
Fistula	1	2	3
Type of SEMS			
Z	2	1	1
UF	4	3	8
NS	0	0	1
Chest pain (%)	5/6 (83.3)^∗^	2/4 (50.0)	3/10 (30.0)^∗^

CRT: chemoradiotherapy; Ce: cervical esophagus; Ut: upper thoracic esophagus; Mt: middle thoracic esophagus; Lt: lower thoracic esophagus; Ae: abdominal esophagus; Z: Cook-Z stent; UF: Ultraflex stent; NS: Niti-S stent; ^∗^*P* = 0.063.

**Table 6:** Relationships of complications and lesion location (*n* = 91 times, number (%)).

	Ce, Ut *n* = 12	Mt *n* = 50	Lt, Ae *n* = 29
Chest pain^※^	4 (33.3)	16 (32.0)	8 (27.6)
Nausea^※^	0	2 (4.0)	0
Hiccup^※^	2 (16.7)	0	0
Gastroesophageal reflux^※^	1 (8.3)	4 (8.0)	5 (17.2)
Recurrent dysphagia^※^	0	4 (8.0)	1 (3.4)
Stent migration^※^	0	0	1 (3.4)
Hemorrhage^※^	0	1 (2.0)	0
Perforation^※^	0	1 (2.0)	1 (3.4)
Airway narrowing^※^	1 (16.7)	1 (2.0)	0
Aspiration pneumonia^※^	0	0	1 (3.4)
Respiratory failure^※^	0	2 (4.0)	0
None	6 (50.0)	27 (54.0)	14 (48.3)

Ce: cervical esophagus; Ut: upper thoracic esophagus; Mt: middle thoracic esophagus; Lt: lower thoracic esophagus; Ae: abdominal esophagus. ^※^There is some overlap.

**Table 7:** Relationships of complications and types of SEMS (*n* = 91 times, number (%)).

	Z *n* = 17	UF *n* = 53	NS *n* = 18	NSu *n* = 3
Chest pain^※^	6 (35.3)	20 (37.7)	2 (11.1)	0
Nausea^※^	0	2 (3.8)	0	0
Hiccup^※^	0	2 (3.8)	0	0
Gastroesophageal reflux^※^	2 (11.8)	8 (15.1)	0	0
Recurrent dysphagia^※^	0	4 (7.5)	1 (5.6)	0
Stent migration^※^	1 (5.9)	0	0	0
Hemorrhaging^※^	0	1 (1.9)	0	0
Perforation^※^	1 (5.9)	0	1 (5.6)	0
Airway narrowing^※^	0	3 (5.7)	0	0
Aspiration pneumonia^※^	0	1 (1.9)	0	0
Respiratory failure^※^	2 (11.8)	0	0	0
None	7 (41.2)	23 (43.4)	14 (77.8)	3 (100)

Z: Cook-Z stent; UF: Ultraflex stent; NS: Niti-S stent; NSu: Niti-S ultrathin stent. ^※^There is some overlap.

**Table 8:** Stent-related fatal cases.

Age (years)	Gender	Location	T4	Reason for insertion	Type of SEMS	Pretreatment	Periods to complication	Complications	Periods from insertion to death (days)
85	Male	Mt, Lt	None	Stenosis	Z	None	During insertion	Perforation	2
49	Female	Mt	None	Stenosis	Z	CRT (40 Gy)	8 hours	Respiratory failure	6
68	Male	Lt, Ae	None	Stenosis	UF	CT	1 day	Aspiration pneumonia	22
81	Male	Ce	Trachea	Stenosis	UF	CRT (65 Gy), GT	3 days	Airway narrowing	25
61	Male	Ut, Mt	Trachea	Fistula	UF	None	15 days	Airway narrowing	22

Ce: cervical esophagus; Ut: upper thoracic esophagus; Mt: middle thoracic esophagus; Lt: lower thoracic esophagus; Ae: abdominal esophagus; Z: Cook-Z stent; UF: Ultraflex stent; CRT: chemoradiotherapy; CT: chemotherapy; GT: genetherapy.

## Abbreviations

SEMS: Self-expandable metallic stent
RT: Radiotherapy
Z stent: Gianturco Z-stent
UF stent: Ultraflex stent
NS stent: Niti-S stent
NSu stent: Niti-S ultrathin stent
CRT: Chemoradiotherapy.
